# Antidiabetic and Anticancer Potentials of *Mangifera indica* L. from Different Geographical Origins

**DOI:** 10.3390/ph16030350

**Published:** 2023-02-24

**Authors:** Rizwan Ahmad, Aljawharah Alqathama, Mohammed Aldholmi, Muhammad Riaz, Ashraf N. Abdalla, Fatema Aljishi, Ebtihal Althomali, Mohd Amir, Omeima Abdullah, Muntathir Ali Alamer, Deema Alaswad, Wala Alsulais, Ahad Alsulays

**Affiliations:** 1 Natural Products and Alternative Medicine, College of Clinical Pharmacy, Imam Abdulrahman Bin Faisal University, Dammam 31441, Saudi Arabia; 2 Department of Pharmacognosy, Faculty of Pharmacy, Umm Al-Qura University, Makkah 21955, Saudi Arabia; 3 Department of Pharmacy, Shaheed Benazir Bhutto University, Sherengal 18050, Khyber-Pakhtunkhwa, Pakistan; 4 Department of Pharmacology and Toxicology, Faculty of Pharmacy, Umm Al-Qura University, Makkah 21955, Saudi Arabia; 5 Department of Pharmaceutical Chemistry, College of Pharmacy, Umm Al-Qura University, Makkah 21955, Saudi Arabia; 6 Prince Sultan Cardiac Center, Al Sulimaniyah 4th, Al Hofuf 36441, Saudi Arabia; 7 College of Clinical Pharmacy, Imam Abdulrahman Bin Faisal University, Dammam 31441, Saudi Arabia

**Keywords:** mango, seed, cytotoxicity, glutathione activity, correlation

## Abstract

Mango fruit is well known for its nutritional and health benefits due to the presence of a plethora of phytochemical classes. The quality of mango fruit and its biological activities may change depending upon the variation in geographical factors. For the first time, this study comprehensively screened the biological activities of all four parts of the mango fruit from twelve different origins. Various cell lines (MCF7, HCT116, HepG2, MRC5) were used to screen the extracts for their cytotoxicity, glucose uptake, glutathione peroxidase activity, and α-amylase inhibition. MTT assays were carried out to calculate the IC_50_ values for the most effective extracts. The seed part from Kenya and Sri Lanka origins exhibited an IC_50_ value of 14.44 ± 3.61 (HCT116) and 17.19 ± 1.60 (MCF7). The seed part for Yemen Badami (119 ± 0.08) and epicarp part of Thailand (119 ± 0.11) mango fruit showed a significant increase in glucose utilization (50 μg/mL) as compared to the standard drug metformin (123 ± 0.07). The seed extracts of Yemen Taimoor seed (0.46 ± 0.05) and Yemen Badami (0.62 ± 0.13) produced a significant reduction in GPx activity (50 μg/mL) compared to the control cells (100 μg/mL). For α-amylase inhibition, the lowest IC_50_ value was observed for the endocarp part of Yemen Kalabathoor (108.8 ± 0.70 μg/mL). PCA, ANOVA, and Pearson’s statistical models revealed a significant correlation for the fruit part vs. biological activities, and seed part vs. cytotoxicity and α-amylase activity (*p* = 0.05). The seed of mango fruit exhibited significant biological activities; hence, further in-depth metabolomic and in vivo studies are essential to effectively utilize the seed part for the treatment of various diseases.

## 1. Introduction

*Mangifera indica* (mango) is one of the most prevalent tropical fruits belonging to the genus *Mangifera*, which involves about 30 species of fruit trees in the Anacardiaceae family [1]. Although mango is known to be native to India, it is now widely cultivated in several countries, Mainly China, Thailand, Indonesia, Mexico, and Pakistan. Several parts of mango, including fruits, leaves, flowers, bark, and roots, have been traditionally used to treat numerous diseases. For instance, an aqueous extract of mango stem bark obtained by decoction has been used for the treatment of diarrhea, menorrhagia, anemia, cutaneous infection, and diabetes [2]. Additionally, it is commonly used in Cuba to enhance the quality of life of cancer patients [3]. Numerous pharmacological studies on mango fruits have been conducted to validate the traditional uses of *Mangifera indica* in the management of diverse diseases. These studies demonstrated that mango fruits have antioxidant, anticancer, and antidiabetic activities [4]. For example, an in vitro study of five Indian mango cultivars showed that different solvent extracts of mango peel have antioxidant, antimicrobial, and anti-inflammatory activities [5]. Regarding the anticancer effect, a recent study revealed that the ethanolic extract of mango peel has antioxidant and cytotoxic effects on lung cancer cell lines [6]. Another study illustrated the antiproliferation effect of the acetone extracts of peel and pulp of different mango cultivars in hepatocellular carcinoma (HepG2) [7]. Furthermore, a group of researchers observed oxidative stress mediated apoptosis induced by ethanolic extract of mango kernels in cultured estrogen receptor-positive breast cancer (MCF-7) [8]. For the antidiabetic potential, mango peel extract exhibited antidiabetic properties via improving antioxidant enzymes in diabetic rats [9]. Additionally, another study found that the methanolic extracts of mango fruits (pulp) offered antidiabetic effects by inhibiting α-amylase and α-glucosidase activity [10]. Moreover, an in vivo study found that long-term administration (21 days) of aqueous and methanolic extract of mango seed effectively reduces blood glucose levels in diabetic rats [11]. Collectively, a considerable amount of the literature proposed the potential application of mango fruit extract in the management of diabetes and cancer. The medicinal benefits of mango are attributed to many bioactive compounds, including polyphenols, terpenoids, carotenoids, and phytosterols [12]. Mangiferin is the prominent potent active constituent of mango with multiple pharmacological properties involving anti-inflammatory, antioxidant, antidiabetic, and anticancer [13]. Mangiferin (1,3,6,7-tetrahydroxyxanthone-C2-β-D- glucoside) is a polyphenol and is mainly isolated from all parts of the *Mangifera indica* tree (fruits, leaves, stem bark, and roots). Different mango cultivars exhibit a wide diversity in mango fruit characteristics (shape, size, color, texture, taste, and aroma) and their phytochemical profiles. Consequently, the bioactivity of each cultivar might be varied. Similarly, various parts of an individual mango fruit (peel, flesh, seed) possess many biological effects due to the variation in the type and quantity of chemical compounds [14]. Indeed, only Nam Doc Mai peel extract showed anti-proliferative effects when a study compared the methanolic extract of peel and pulp from three mango varieties (Irwin, Nam Doc Mai, and Kensington Pride) for their growth-inhibitory potentials on MCF-7 human breast cancer cells. Overall, fruit parts and habitat differences are essential factors to consider when evaluating medicinal plants’ bioactivity. To the best of our knowledge, this is a first-time study to evaluate the anticancer and antidiabetic activities of an aqueous extract of four separate parts (epi-, meso-, endocarp, and seed) of eight mango fruits from different geographical origins (Indian, Egyptian, Pakistani, Yemen, Thailand, Sri Lanka, Kenya, and Vietnam). In addition, it assesses the correlation between the Mangiferin amount and the observed biological activities. To illustrate, the cytotoxicity potential of the mango fruit extract was tested in selected cancer cell lines (Human breast adenocarcinoma, Human colorectal carcinoma, and Hepatocellular carcinoma) and normal human fetal lung fibroblast. Also, the effect of mango extracts on the glutathione peroxidase enzyme was assessed. Lastly, the antidiabetic activity was investigated by the inhibition of α-amylase, as well as the glucose uptake capacity in hepatocellular carcinoma.

## 2. Results

### 2.1. Determination of the Cytotoxic Activities

The cytotoxicity was observed in the range of (%) 34–91 for HCT116 and 34–98 for MCF7. The extracts with cytotoxic effect >50% were selected for further MTT assay in order to find their IC_50_ values (Table 1): India Badami (large green) seed (HCT116 (37 ± 0.11), MCF7 (37 ± 0.11)), India Totapuri (long green) seed (HCT116 (45 ± 0.16), MCF7 (34 ± 0.01)), Egypt (reddish green) epicarp (HCT116 (41 ± 0.12), MCF7 (38 ± 0.08)), Egypt (reddish green) seed (HCT116 (50 ± 0.11), MCF7 (47 ± 0.07)), Kenya (large round red) seed (HCT116 (34 ± 0.08), MCF7 (38 ± 0.11)), Sri Lanka (large yellowish green) seed (HCT116 (39 ± 0.12), MCF7 (34 ± 0.05)), Thailand (large green) seed (HCT116 (35 ± 0.15), MCF7 (36 ± 0.01)), Vietnam (large yellowish green) seed (HCT116 (42 ± 0.17), MCF7 (39 ± 0.05)), Yemen Badami (large yellow) seed (HCT116 (42 ± 0.17), MCF7 (39 ± 0.03)), Yemen Kalabathoor (large round red) endocarp (HCT116 (50 ± 0.11), MCF7 (43 ± 0.01)), Yemen Kalabathoor (large round red) seed (HCT116 (48 ± 0.13), MCF7 (40 ± 0.02)), Yemen Taimoor (large yellowish green) seed (HCT116 (42 ± 0.21), MCF7 (38 ± 0.05)), and Yemen Taimoor (small reddish green) seed (HCT116 (36 ± 0.12), MCF7 (39 ± 0.07)).

Six different concentrations with the addition of an MRC5 cell line were utilized for selectivity determination. The mango fruit extract from Kenya (large round red) with seed part was the most effective against HCT116 (14.44 ± 3.61), whereas the extract from Sri Lanka (large yellowish green) seed revealed a significant effect against MCF7 cell line (17.19 ± 1.60). Likewise, the extracts from Kenya (large round red) seed (14.44 ± 3.61 and 25.96 ± 1.08), Vietnam (large yellowish green) seed (26.54 ± 1.10 and 20.17 ± 1.24), and Thailand (large green) seed (20.01 ± 0.88 and 24.63 ± 2.53) showed more selective cytotoxicity towards MCF7 and HCT116 cell lines respectively, as compared to MRC5 cell line. The Sri Lankan (large yellowish green) seed extract exhibited a comparatively more significant effect against HCT116 (20.53 ± 1.56), MCF7 (17.19 ± 1.60), and MRC5 cell line (20.79 ± 1.59). The data are shown in Table 2.

### 2.2. The effect of Extracts on Glucose Uptake Using HepG2

The cell viability was initially evaluated using MTT assay to check for the cytotoxic effect of extracts (100 μg/mL) on HepG2 followed by confirmation of the actual dose for glucose utilization assay. The MTT assay confirmed a lack of cytotoxicity (100 μg/mL) for all the extracts except the following: Yemen Taimoor (small reddish green) seed, Thailand (large green) seed, Kenya (large round red) seed, Vietnam (large yellowish green) seed, Yemen Kalabathoor (large round red) endocarp, Yemen Badami (large yellow) seed, Yemen Taimoor (large yellowish green) seed, and Yemen Kalabathoor (large round red) seed, which showed no cytotoxicity at 50 μg/mL (data not shown). The cells treated with the extracts of Thailand (large green) epicarp (119 ± 0.11), Kenya (large round red) mesocarp (118 ± 0.07), Yemen Taimoor (large yellowish green) epicarp (115 ± 0.05), Yemen Badami (large yellow) endocarp (114 ± 0.06), Indian Alphonso (small round green) mesocarp (114 ± 0.05), Kenya (large round red) endocarp (112 ± 0.06), Thailand (large green) mesocarp (110 ± 0.05), Thailand (large green) endocarp (109 ± 0.05), Yemen Badami (large yellow) epicarp (108 ± 0.04), and Kenya (large round red) epicarp (105 ± 0.02) exhibited significant (*p* < 0.05) increase in glucose uptake and utilization in HepG2 cells (100 μg/mL). The extract for Yemen Badami (large yellow) seed (119 ± 0.08) showed a significant increase in glucose utilization at 50 μg/mL, whereas the results for both extracts of Yemen Badami (large yellow) seed (119 ± 0.08) and Thailand (large green) epicarp (119 ± 0.11) were comparable with the result of control (metformin = 123 ± 0.07) used in this study (Table 1).

### 2.3. The Effect of Extracts on Glutathione Peroxidase Activity (GPx) Using HepG2

For relative GPx activity, MTT-assisted cell viability was evaluated as described earlier. The tested concentration (100 μg/mL) showed a lack of cytotoxic effect for all the extracts except the following: Yemen Taimoor (small reddish green) seed, Thailand (large green) seed, Kenya (large round red) seed, Vietnam (large yellowish green) seed, Yemen Kalabathoor (large round red) endocarp, Yemen Badami (large yellow) seed, Yemen Taimoor (large yellowish green) seed, and Yemen Kalabathoor (large round red) seed where no sign of cytotoxicity was observed at 50 μg/mL (data not shown). A significant (*p* < 0.05) decrease in GPx activity was observed for cells treated with the extracts (100 μg/mL) of Egypt (reddish green) endocarp (0.25 ± 0.13), Thailand (large green) mesocarp (0.36 ± 0.12), Thailand (large green) endocarp (0.42 ± 0.11), Yemen Taimoor (large yellowish green) epicarp (0.53 ± 0.04), India Totapuri (long green) seed (0.53 ± 0.14), Sri Lanka (large yellowish green) seed (0.54 ± 0.23), Yemen Taimoor (large yellowish green) endocarp (0.57 ± 0.32), Yemen Kalabathoor (large round red) mesocarp (0.60 ± 0.17), and Sri Lanka (large yellowish green) epicarp (0.61 ± 0.11), as compared to the control cells. Extracts of Yemen Taimoor (small reddish-green) seed (0.46 ± 0.05) and Yemen Badami (large yellow) seed (0.62 ± 0.13) showed a significant reduction for GPx activity at a dose of 50 μg/mL. The data for GPx activity are shown in Table 1.

### 2.4. The Effect of Extracts on the α-Amylase Activity

The initial screening (500 μg/mL) for α-amylase activity revealed inhibition of >50% for Yemen Kalabathoor (large round red) endocarp (60 ± 0.05), Vietnam (large yellowish green) seed (58 ± 0.07), India Badami (large green) seed (55 ± 0.13) extracts, and Yemen Taimoor (small reddish-green) seed (54 ± 0.06), as shown in Table 1. These extract samples were further investigated at six different concentrations for the determination of the IC_50_ values. The extract for Yemen Kalabathoor (large round red) endocarp showed a comparatively low IC_50_ value (μg/mL) of 108.8 ± 0.70, as compared to the standard drug acarbose (78.41 ± 0.67 μg/mL). The IC_50_ values for the tested extracts are shown in Table 3.

### 2.5. Statistical Analysis

The results are expressed with a mean (±standard deviation (SD)) from at least three independent experiments. The data were analyzed for statistical significance between the treated and control group with the help of GraphPad Prism V-9.2.0 (GraphPad, San Diego, CA, USA) at *p* < 0.05, whereas for correlations and component analysis, SPSS (statistical package for the social sciences) V 22.0 was used.

#### 2.5.1. Descriptive Analysis

The descriptive analysis showed a range with a mean (±SD) of 34–91 and 68.6 (±17.84) for HCT116, 34–98, and 64.81 (±19.28) for MCF7, 95–119, and 102.02 (±6.67) for glucose uptake, 0.25–1.30 and 0.722 (±0.21) for GPx, and 1–60 and 18.70 (±15.16) for α-amylase (Table 1).

#### 2.5.2. Correlation Analysis

##### Pearson’s Correlation

The statistical analysis for Pearson’s analysis showed a significant correlation between geographical origin and MG amount (−0.603, *p* = 0.000). With regard to biological activities, a significant correlation was observed for the fruit part vs. HT116 (−0.550, *p* = 0.000), MCF7 (−0.442, *p* = 0.002), and α-amylase activity (0.315, *p* = 0.029). Glucose uptake and GPx activities do not correlate with the geographical origin, fruit part, MG amount, or biological activities (Table 4).

##### Component Analysis for Variance

The principal component analysis (PCA), an eigenvalue-based statistical tool, is employed to reduce the dimensions of a large dataset into principal components based on %variability. The PCA maximizes the variance for uncorrelated variables based on an individual and cumulative variance, with an impactful display of the relevant correlated variables in the same component. Herein, three components, PC1 (33.09%), PC2 (20.48%), and PC3 (14.65%), with a cumulative % variability of 68.23, were observed. For PC1 with major % variability, the variables presented were fruit part (−0.70), HCT116 (0.92), MCF7 (0.87), and α-amylase activity (−0.60), geographical origin (−0.91) and MG amount (0.83) were observed in PC2, whereas glucose uptake (0.62) and GPx activity (0.79) were observed in PC3, i.e., with the least % variability. The KMO-Bartlett’s test of sphericity showed a high *X*^2^-value of 129.78 with a significance value of 0.00 (*p* = 0.05) the data for PCA are shown in Table 5.

**Figure 1:**
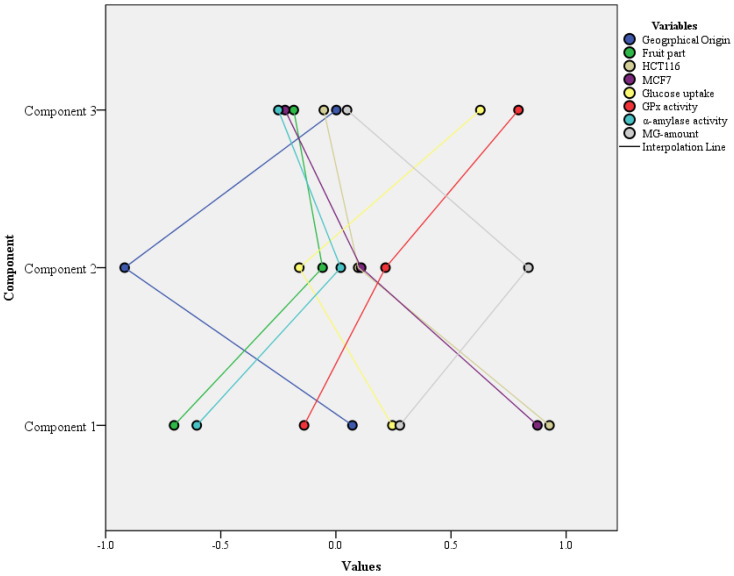
Components with respective variables distribution for mango extract activities.

A graphical presentation of the components observed with their corresponding eigenvalues is shown in Figure 1.

##### Variables Correlation with ANOVA

ANOVA with inter- and intra-correlation for the data variables also confirmed the outcomes from Pearson’s correlation and PCA analysis. The correlation of geographical origin with MG amount (−0.603), as well as fruit part with HCT116 (−0.550), MCF7 (−0.442), and α-amylase activity (0.315), was observed to be significant (0.00) with a high F-value of 627.81. The correlation for within and between groups, along with the sum of squares and mean square values, is shown in Table 6 (*p* = 0.05).

## 3. Discussion

It is a well-known fact that variations in the geographical origin of a plant or fruit significantly affect its quality in terms of phytochemical profile and biological activities. The main aim of this study was to compare the quality variation among mango fruits obtained from different geographical origins. Mango fruits from eight different cultivars were collected and processed for green extraction and characterization of phytochemical profile based on MG amount in four parts of each mango fruit (epi-, endo-, mesocarp, and seed). The details regarding the novel green extraction and LCMSMS characterization for MG amount in these four parts of eight cultivars are described in our previous study [15]. The extracts from these mango fruit samples were screened for biological activities consisting of cytotoxicity (HCT116, MCF7, MRC5 cell lines), glucose uptake assay and GPx activity in HepG2 cells, and α-amylase activity. Statistical models were created to establish a correlation between the phytochemicals present in the mango fruit with the biological activities tested.

For cytotoxicity (HCT116, MCF7, MRC5), a general followed by an in-depth screening was performed to determine the IC_50_ values. The extract samples with <50% of cell viability were studied further at six different concentrations using an additional cell line of MRC5. In general, the seed parts of these mango cultivars were observed with considerable cytotoxic potential. The seed extract from Kenya mango (large and round) was seen to be more cytotoxic towards the tested cell lines (HCT116, MCF7) in general screening, whereas the MTT assay for selectivity against the three cell lines (HCT116, MCF7, and MRC5) suggested Sri Lanka (large yellowish green) seed extract with the lowest IC_50_ value followed by Thailand (large green) seed extract. Though various cytotoxic studies have been reported for mango fruit extract including the following: aqueous flesh extract [16], peel and flesh for Australian cultivars [14], peel extract from six Brazilian cultivars [17,18], seed extract [8,19,20] as well as skin and flesh extract [21], this is a first-time study to report a comprehensive characterization for the whole mango fruit parts from eight different cultivars. With regard to phytochemicals in mango fruit, a good amount of MG was observed in all the fruit parts of these mango cultivars. Though seeds were reported with an average amount of MG, the epi- and mesocarp parts of the mango fruit presented the highest MG amount. MG and MG gallate [22], along with phenols and oleanolic acid, have been reported to possess significant cytotoxic potential [6]. The 48 extracted samples from the eight cultivars exhibited a significant (*p* < 0.05) increase in glucose uptake and utilization in HepG2 cell lines. The epicarps (Thailand (large green), Yemen Taimoor (large yellowish green), Yemen Badami (large yellow), Kenya (large round red)), endocarps (Yemen Badami (large yellow), Kenya (large round red), Thailand (large green)), and mesocarps (Kenya (large round red), Indian Alphonso (small round green), Thailand (large green)) parts were more effective as compared to the seeds of the mango fruits (100 μg/mL). In addition, the extracts of Yemen Badami (large yellow) seed and Thailand (large green) epicarp revealed a significant increase in glucose uptake at 50 μg/mL. As mentioned earlier, the MG amount was found more in the mentioned parts of these different cultivars fruit samples, epicarp, and mesocarp in particular. Mangiferin has been reported to promote glucose utilization and metabolism in a dose-dependent manner [23], promote PPARα-induced free fatty acids metabolism in HepG2 cells [24], enhance the utilization of peripheral glucose [23], and decrease β-cells apoptosis [25]. The GPx (glutathione peroxidase) enzyme prevents cell damage via the reduction of free radicals [26,27]; however, with an increase in free radicals in various disease conditions, an increase in GPx level is usually witnessed, which returns to its normal level with the use of specific therapeutic agents [28,29]. An elevated level of GPx prevents oxidative damage and inflammation, but this may block apoptotic cell death, resulting in a higher survival rate for the altered cells. This shows a complex role for GPx in the development and progression of cancer due, in part, to its role in the modulation of intracellular ROS [29]. The extracts were tested for a potential role in GPx enzyme inhibition. Herein again, the epicarps (Yemen Taimoor (large yellowish green), Sri Lanka (large yellowish green)), mesocarps (Thailand (large green), Yemen Kalabathoor (large round red)), and endocarps (Egypt (reddish green), Thailand (large green), Yemen Taimoor (large yellowish green)) parts of these mango fruits samples showed considerable reduction of GPx activity in HepG2 cells. A significant activity reduction (as compared to the positive control, i.e., tannic acid) was recorded for the extracts of Egypt (reddish green) endocarp and Thailand (large green) mesocarp; however, in vivo assays are required to further investigate and confirm the effect of these extracts on GPx level. The more MG amount in the mango fruits epicarp and mesocarp may be suggested to play a role in GPx restoration and its normalization to the normal level, as reported previously [30,31]. One of the investigative targets for diabetes management is the control of postprandial glucose levels via inhibition of the carbohydrate-digesting enzyme, i.e., α-amylase in the intestine [32,33]. In order to find the antidiabetic potential for the mango fruit parts, α-amylase inhibition activity was investigated. The general screening results suggested considerable activity for the extracts of Yemen Kalabathoor (large round red) endocarp, Vietnam (large yellowish green) seed, India Badami (large green) seed, and Yemen Taimoor (small reddish-green) seed. The extracts from these cultivars were studied further for selectivity (i.e., being selectively cytotoxic to cancer cells and less cytotoxic to normal cells. Extracts with lower cytotoxicity to normal cells are considered a selective extract against cancer cell lines) and IC_50_ value determination, where the endocarp from Yemen Kalabathoor (large round red) exhibited considerably low IC_50_ values when compared to the standard drug acarbose. The amount of MG in the endocarp is reported to be higher compared to seeds; hence, MG, along with some other constituents, may be responsible for the α-amylase activity. Previous studies have confirmed the α-amylase inhibitory role of mango peel-extracted MG [34,35,36]. Furthermore, in vivo studies on mango peel extract also supported the antidiabetic potential for MG [9,34,37]. However, phenolic compounds may also play a vital role as suppressors of postprandial hyperglycemia at different concentrations [38].

The outcomes of the biological activities suggest the presence of more than one bioactive phytochemical in mango fruit and its different parts. Albeit MG was observed with an average amount in all the 48 samples extracted for mango fruit, higher in epicarp and mesocarp parts, a systemic in-depth metabolomic analysis may be more useful to obtain a broader profile of the phytochemicals present in different varieties of the mango fruit (prone to variation based on the change in geography and other factors) vs. the effectiveness in biological activities of these samples. Moreover, the active phytochemicals (based on biological assays) need to be isolated and tested further via in vivo studies in different concentrations. The isolated compounds might show lower activity compared to the mixture of compounds in the extract.

The statistical models were applied to show the correlation among the biological activities of the different parts of the mango fruit from different cultivars. Pearson’s test revealed a significant correlation between the geographical origin vs. MG amount and fruit part vs. cytotoxicity (HCT116, MCF7) and α-amylase activity. Glucose uptake assay and GPx inhibition showed no correlation with the mentioned biological activities. For the fruit part, it was the seed part of the fruit that appeared as the most effective in cytotoxicity and α-amylase inhibitory activity. Likewise, the PCA analysis showed more %variability (intra-correlation) for PC1 with components of fruit part, HCT116, MCF7, and α-amylase. Geographical origin and MG amount were placed in PC2, whereas glucose uptake and GPx activities were grouped in PC3 with the least % variability, suggesting a more significant correlation for the seed fruit part with cytotoxicity and α-amylase activity. The ANOVA with inter- and intra-correlation for the dataset further confirmed an alike significant correlation with a high F-value as reported for Pearson’s and PCA analysis. The outcomes of the statistical model suggest a prominent role for the seed part of the mango fruit in various biological activities. It is noteworthy to mention that it is the first time to report the mangiferin-based characterization of the different mango fruit parts (epi-, endo-, mesocarp, and seed) from eight different cultivars (48 samples) with an extensive biological screening of cytotoxicity (HCT116, MCF7, MRC5), glucose utilization (HepG2), GPx inhibition (HepG2), and α-amylase inhibition. None of the literature is available to be compared with the outcomes herein. The authors suggest a detailed metabolomic or phytochemical analysis to establish a prominent role in a series of biological activities for the seed part of the mango fruit. These in vitro studies may be followed by appropriate in vivo pharmacological experiments in order to confirm the role of mango seed, which may become a good source of phytochemicals, food products, and nutraceuticals to be utilized for the management and cure of various ailments.

## 4. Materials and Methods

### 4.1. Collection and Preparation of Samples

Fresh mango fruit varieties from different geographical origins (Indian, Egyptian, Pakistani, Yemen, Thailand, Sri Lanka, Kenya, and Vietnam) were collected from local markets at Khobar, Eastern Province, Kingdom of Saudi Arabia. A green ultrasonic-assisted extraction (using water as a solvent at a temperature of 40 °C) was performed for four different parts (epi-, meso-, endocarp, and seed) of every individual mango fruit, as reported in our previous study [15].

### 4.2. Cells and Microorganisms

Human breast adenocarcinoma (MCF7: ATCC-HTB22); Human colorectal carcinoma (HCT116: ATCC-CCL247); Hepatocellular carcinoma (HepG2: ATCC-HB8065); normal human fetal lung fibroblast (MRC5: ATCC-CCL171).

### 4.3. Chemicals and Reagents

Dimethyl sulfoxide (DMSO from Sigma Aldrich, St. Louis, MO, USA); RPMI-1640 (Roswell Park Memorial Institute Medium), DMEM (Dulbecco’s Modified Eagle Media), FBS (fetal bovine serum), penicillin (10,000 units/mL), and streptomycin (10,000 µg per mL) from Gibco, Life Technologies, Carlsbad, CA, USA; MTT assay reagent and α-amylase reagent Sigma Aldrich); Glucose uptake assay Kit (GAGO20) from Sigma Aldrich, St Louis, MO, USA; glutathione peroxidase Kit (ab102530) from Abcam, Cambridge, UK; doxorubicin (98.0–102.0% (HPLC)) from Sigma Aldrich and metformin (97%) from Merck, whereas Microplate reader used was from BIORAD, PR 4100, Hercules, CA, USA.

### 4.4. Characterization and Standardization of the Extracts

The extracts were quantified for Mangiferin amount (MG amount) in all the mango fruit samples. UPLCMS/MS was applied to develop and validate a green, efficient, and fast analytical method for MG quantification [15]. Though reported in our previous study, the MG amount found in these samples is presented in the table below for ease of understanding of the correlation.

### 4.5. Cell Culture

All cell lines were cultured and maintained according to the procedure reported by Ahmad et al. [39].

### 4.6. Determination of Cytotoxicity and Selectivity

The cytotoxicity of the extracts was evaluated by MTT assay, as previously reported [40] and the already reported method of Ahmad et al. [39].

### 4.7. Glucose Uptake Assay

Odeyemi et al. modified method was used for glucose uptake assay [39,41].

### 4.8. Determination of Glutathione Peroxidase Activity

The assay was performed according to the manufacturer’s protocol, and our group reported procedures [26,28,39].

### 4.9. α-Amylase Inhibition Activity

The inhibitory activity of α-amylase was ascertained as described by Quan et al. [42]. The extracts were initially tested for 500 μg/mL, extracts that inhibited the enzyme were further evaluated for 1000, 500, 100, 50, 25, and 10 μg/mL, with acarbose as a positive control [39]. Absorbance was measured using a multi-plate reader at 550 nm for each well, calculating the percentage of inhibition utilizing the following equation:

% inhibition = (A − C/B − C) × 100,

where A = the absorbance of the reaction mixture in the presence of the extract, B = the absorbance of the mixture without the enzyme, and C = the absorbance of the reaction mixture in the absence of any extract.

## 5. Conclusions

This study investigated the quality of eight different cultivars of mango fruit in terms of the biological activities of the fruit parts. All parts of the mango fruit showed cytotoxicity, glucose uptake, GPx, and α-amylase inhibition. The statistical model suggested a significant correlation with most activities attributable to the seed part of the mango fruit. MG was correlated to the screened biological activities; however, a comprehensive phytochemical characterization with advanced in vivo studies and clinical trials may further confirm the role of mango fruit for the cure of diseases in the form of food products, nutraceuticals, or isolated medicinal compounds. The study assessed quality variation among different mango cultivars and established a correlation between phytochemistry and the biological activity of mango fruit and its parts from different geographical origins.

## Figures and Tables

**Table 1:** Cytotoxic (MTT 48 h, % of cell viability ± SD), glucose uptake in HepG2 (48 h, % of glucose utilization ± SD), GPx activity in HepG2 cells (48 h, relative GPx activity compared to the control cells ± SD), and α-Amylase inhibitory activity (% of enzyme inhibition ± SD μg/mL) with respective descriptive statistics (* *p* = 0.05, ** *p* = 0.01, *** *p* = 0.001).

Original Code		HCT116	MCF7	Glucose Uptake	GPx Activity	α-Amylase Inhibition	MG Amount (mg/10 g)
India Badami (large green) epicarp		91 ± 0.25	71 ± 0.03	101 ± 0.04	0.50 ± 0.31	7 ± 0.04	0.81
India Badami (large green) endocarp		78 ± 0.11	73 ± 0.16	98 ± 0.02	0.90 ± 0.16	10 ± 0.09	0.44
India Badami (large green) mesocarp		72 ± 0.38	65 ± 0.21	99 ± 0.01	0.61 ± 0.23	16 ± 0.01	1.07
India Badami (large green) seed		37 ± 0.11	37 ± 0.01	96 ± 0.08	0.90 ± 0.19	55 ± 0.13	0.51
India Totapuri (long green) epicarp		63 ± 0.11	81 ± 0.06	99 ± 0.06	0.72± 0.21	3 ± 0.03	1.10
India Totapuri (long green) endocarp		86 ± 0.12	80 ± 0.01	99 ± 0.03	0.81 ± 0.12	3 ± 0.04	1.10
India Totapuri (long green) mesocarp		74 ± 0.09	67 ± 0.07	99 ± 0.03	0.92 ± 0.13	9 ± 0.12	1.49
India Totapuri (long green) seed		45 ± 0.16	34 ± 0.01	101 ± 0.06	0.53 ± 0.14 **	4 ± 0.04	1.39
Indian Alphonso (small round green) epicarp		74 ± 0.24	73 ± 0.03	100 ± 0.04	1.09 ± 0.17	13 ± 0.04	1.25
Indian Alphonso (small round green) endocarp		81 ± 0.14	82 ± 0.02	99 ± 0.04	0.72 ± 0.33	4 ± 0.03	1.81
Indian Alphonso (small round green) mesocarp		80 ± 0.16	74 ± 0.03	114 ± 0.05 **	0.96 ± 0.23	39 ± 0.12	2.09
Indian Alphonso (small round green) seed		70 ± 0.37	58 ± 0.30	100 ± 0.06	0.57 ± 0.32	30 ± 0.08	0.99
Egypt (reddish green) epicarp		41 ± 0.12	38 ± 0.08	98 ± 0.05	0.63 ± 0.23	21 ± 0.16	0.65
Egypt (reddish green) endocarp		83 ± 0.12	95 ± 0.02	99 ± 0.02	0.25 ± 0.13 ***	9 ± 0.03	0.87
Egypt (reddish green) mesocarp		84 ± 0.16	83 ± 0.15	99 ± 0.05	0.59 ± 0.37	15 ± 0.11	0.78
Egypt (reddish green) seed		50 ± 0.11	47 ± 0.07	104 ± 0.06	0.85 ± 0.24	20 ± 0.07	0.47
Kenya (large round red) epicarp		67 ± 0.13	62 ± 0.09	105 ± 0.02 *	0.70 ± 0.26	4 ± 0.02	0.56
Kenya (large round red) endocarp		88 ± 0.13	87 ± 0.09	112 ± 0.06 *	0.89 ± 0.21	1 ± 0.08	0.57
Kenya (large round red) mesocarp		71 ± 0.36	39 ± 0.36	118 ± 0.07 *	1.30 ± 0.23	18 ± 0.15	0.76
Kenya (large round red) seed		34 ± 0.08	38 ± 0.11	98 ± 0.10	0.63 ± 0.27	33 ± 0.09	0.53
Sri Lanka (large yellowish green) epicarp		75 ± 0.15	61 ± 0.10	99 ± 0.05	0.61 ± 0.11 **	12 ± 0.05	0.69
Sri Lanka (large yellowish green) endocarp		82 ± 0.12	82 ± 0.01	102 ± 0.05	0.72 ± 0.21	1 ± 0.01	0.76
Sri Lanka (large yellowish green) mesocarp		81 ± 0.18	80 ± 0.06	97 ± 0.02	0.62 ± 0.28	15 ± 0.03	0.70
Sri Lanka (large yellowish green) seed		39 ± 0.12	34 ± 0.05	96 ± 0.02	0.54 ± 0.23 *	8 ±0.02	0.82
Thailand (large green) epicarp		75 ± 0.08	68 ± 0.17	119 ± 0.11 *	0.86± 0.18	21 ± 0.08	0.88
Thailand (large green) endocarp		80 ± 0.08	73 ± 0.04	109 ± 0.05 *	0.42 ± 0.11 ***	19 ± 0.03	0.87
Thailand (large green) mesocarp		87 ± 0.11	89 ± 0.02	110 ± 0.05 *	0.36 ± 0.12 **	41 ± 0.11	0.66
Thailand (large green) seed		35 ± 0.15	36 ± 0.01	101 ± 0.07	0.98± 0.18	16 ± 0.13	0.54
Vietnam (large yellowish green) epicarp		72 ± 0.20	70 ± 0.06	100 ± 0.05	0.99 ± 0.28	9 ±0.04	0.61
Vietnam (large yellowish green) endocarp		84 ± 0.21	75 ± 0.16	95 ± 0.06	0.88 ± 0.33	8 ±0.03	0.91
Vietnam (large yellowish green) mesocarp		87 ± 0.12	80 ± 0.06	100 ± 0.07	0.55± 0.28	28 ± 0.08	0.89
Vietnam (large yellowish green) seed		42 ± 0.17	39 ± 0.05	96 ± 0.03	0.72 ± 0.26	58 ± 0.07	0.47
Yemen Badami (large yellow) epicarp		80 ± 0.21	74 ± 0.09	108 ± 0.04 *	0.79 ± 0.19	14 ± 0.03	0.97
Yemen Badami (large yellow) endocarp		77 ± 0.14	80 ± 0.07	114 ± 0.06 *	0.63 ± 0.38	5 ± 0.03	0.76
Yemen Badami (large yellow) mesocarp		90 ± 0.18	64 ± 0.03	101 ± 0.01	0.72± 0.40	15 ± 0.04	0.94
Yemen Badami (large yellow) seed		42 ± 0.17	39 ± 0.03	119 ± 0.08 *	0.62 ± 0.13 **	31 ± 0.07	0.75
Yemen Kalabathoor (large round red) epicarp		76 ± 0.10	84 ± 0.13	99 ± 0.04	0.70± 0.24	14 ± 0.02	1.52
Yemen Kalabathoor (large round red) endocarp		50 ± 0.11	43 ± 0.01	96 ± 0.05	0.57 ± 0.32	60 ± 0.05	0.77
Yemen Kalabathoor (large round red) mesocarp		67 ± 0.16	62 ± 0.06	95 ± 0.04	0.60 ± 0.17 *	13 ± 0.08	1.42
Yemen Kalabathoor (large round red) seed		48 ± 0.13	40 ± 0.02	97 ± 0.03	0.77 ± 0.19	28 ± 0.11	0.81
Yemen Taimoor (large yellowish green) epicarp		74 ± 0.17	57 ± 0.15	115 ± 0.05 **	0.53 ± 0.04 ***	5 ± 0.15	0.95
Yemen Taimoor (large yellowish green) endocarp		77 ± 0.12	84 ± 0.24	97 ± 0.02	0.63 ± 0.14 *	12 ± 0.07	0.58
Yemen Taimoor (large yellowish green) mesocarp		87 ± 0.03	98 ± 0.17	101 ± 0.03	0.80 ± 0.20	34 ± 0.13	0.84
Yemen Taimoor (large yellowish green) seed		42 ± 0.21	38 ± 0.05	98 ± 0.09	1.25 ± 0.20	12 ± 0.09	0.82
Yemen Taimoor (small reddish green) epicarp		73 ± 0.10	62 ± 0.13	98 ± 0.02	0.84 ± 0.12	19 ± 0.09	0.96
Yemen Taimoor (small reddish green) endocarp		80 ± 0.09	92 ± 0.08	98 ± 0.02	0.89 ± 0.25	15 ± 0.01	1.01
Yemen Taimoor (small reddish green) mesocarp		85 ± 0.15	84 ± 0.02	102 ± 0.07	0.58 ± 0.36	17 ± 0.10	0.79
Yemen Taimoor (small reddish green) seed		36 ± 0.12	39 ± 0.07	97 ± 0.05	0.46 ± 0.05 ***	54 ± 0.06	0.61
Standard		-	-	Metformin (123 ± 0.07 **)	Tannic acid (0.44 ± 0.13 ***)	-	-
Descriptive statistics							
Descriptive	HCT116	MCF7	Glucose uptake		GPx activity	α-Amylase inhibition	
Minimum	34	34	95		0.25	1	
Maximum	91	98	119		1.30	60	
Mean	68.58	64.81	102.02		0.72	18.70	
Standard deviation	17.84	19.28	6.67		0.21	15.16	

**Table 2:** Cytotoxicity and selectivity of the selected extracts (MTT 48 h, IC_50_ ± SD μg/mL).

Geographical Origin	HCT116	MCF7	MRC5
Egypt (reddish green) seed	87.54 ± 3.03	70.99 ± 2.74	96.62 ± 2.33
Yemen Kalabathoor (large round red) endocarp	96.63 ± 2.73	74.83 ± 1.71	72.86 ± 1.65
Yemen Kalabathoor (large round red) seed	76.33 ± 1.95	36.83 ± 1.70	69.91 ± 2.50
Vietnam (large yellowish green) seed	26.54 ± 1.10	20.17 ± 1.24	55.60 ± 1.07
Kenya (large round red) seed	14.44 ± 3.61	25.96 ± 1.08	42.57 ± 1.76
Yemen Taimoor (large yellowish green) seed	43.66 ± 1.92	34.69 ± 1.94	39.30 ± 2.14
Egypt (reddish green) epicarp	51.48 ± 3.72	33.11 ± 2.51	39.09 ± 3.63
India Badami (large green) seed	22.99 ± 2.00	26.60 ± 0.96	29.95 ± 2.64
Yemen Badami (large yellow) seed	28.76 ± 2.90	30.35 ± 1.06	27.85 ± 1.63
Thailand (large green) seed	20.01 ± 0.88	24.63 ± 2.53	27.47 ± 3.54
Yemen Taimoor (small reddish green) seed	18.16 ± 2.92	25.76 ± 2.64	26.00 ± 3.35
Sri Lanka (large yellowish green) seed	20.53 ± 1.56	17.19 ± 1.60	20.79 ± 1.59
India Totapuri (long green) seed	56.47 ± 2.55	37.44 ± 1.02	100.4 ± 1.43
Doxorubicin	4.19 ± 1.23	3.11 ± 1.34	6.90 ± 0.95

**Table 3:** α-Amylase inhibitory activity of the selected extracts (IC_50_ ± SD μg/mL).

Geographical Origin	IC_50_
Yemen Taimoor (small reddish-green) seed	190.5 ± 2.35
Vietnam (large yellowish green) seed	128.4 ± 1.25
Yemen Kalabathoor (large round red) endocarp	108.8 ± 0.70
India Badami (large green) seed	202.5 ± 2.80
Acarbose	78.41 ± 0.67

**Table 4:** Pearson’s correlation analysis for the mango extracts activities.

	Geographical Origin	Fruit Part	HCT116	MCF7	Glucose Uptake	GPx Activity	α-Amylase Activity	MG Amount
Fruit part	0.000 1.000	1						
HCT116	−0.044 0.764	−0.550 0.000	1					
MCF7	−0.060 0.686	−0.442 0.002	0.883 0.000	1				
Glucose uptake	0.074 0.615	−0.182 0.216	0.198 0.177	0.058 0.693	1			
GPx activity	−0.160 0.277	−0.032 0.828	−0.079 0.591	−0.153 0.299	0.108 0.466	1		
α-amylase activity	−0.089 0.548	0.315 0.029	−0.430 0.002	−0.373 0.009	−0.079 0.592	−0.109 0.460	1	
MG amount	−0.603 0.000	−0.237 0.104	0.280 0.054	0.248 0.089	0.047 0.751	0.069 0.639	−0.198 0.177	1

**Table 5:** Principal component analysis with KMO and Bartlett’s test for mango extracts activities.

Factors	PC1	PC2	PC3	Kaiser-Meyer-Olkin and Bartlett’s Test		
Geographical origin	0.072	−0.918	0.002	KMO Measure of Sampling Adequacy		0.60
Fruit part	−0.703	−0.058	−0.183	Bartlett’s Test of Sphericity	Approx. Chi-Square	129.78
HCT116	0.928	0.097	−0.053		df	28
MCF7	0.875	0.110	−0.221		Sig.	0.00
Glucose uptake	0.245	−0.159	0.627			
GPx activity	−0.138	0.216	0.793			
α-amylase activity	−0.604	0.021	−0.250			
MG amount	0.278	0.837	0.049			
Individual variance (%)	33.092	20.487	14.653			
Cumulative variance (%)	33.092	53.579	68.232			

**Table 6:** Inter- and intra-correlation analysis with ANOVA for mango extract activities.

	Geographical Origin	Fruit Part	HCT116	MCF7	Glucose Uptake	GPx Activity	α-Amylase Activity
Fruit part	0.000						
HCT116	−0.044	−0.550					
MCF7	−0.060	−0.442	0.883				
Glucose uptake	0.074	−0.182	0.198	0.058			
GPx activity	−0.160	−0.032	−0.079	−0.153	0.108		
α-amylase activity	−0.089	0.315	−0.430	−0.373	−0.079	−0.109	
MG amount	−0.603	−0.237	0.280	0.248	0.047	0.069	−0.198
ANOVA table							
		Sum of Squares		Mean Square		F	Sig
Between groups		6634.99		141.17		627.81	0.00
Within groups	Between Items	525,689.83		75,098.54			
	Residual	39,354.55		119.61			
	Total	565,044.39		1681.68			

## Data Availability

The data used to generate the outcomes is completely presented in this document.
